# A Peacock That Does Not Show Its Feathers Is Not Much Different From a Turkey: On Being a Physician Executive for 25 Years

**DOI:** 10.1093/geroni/igaa057.125

**Published:** 2020-12-16

**Authors:** Saraswathy Battar

**Affiliations:** Michael E. DeBakey VA Medical Center, Houston, Texas, United States

## Abstract

Compelling common threads in the fabric of being an academic and clinical geriatrician, VA health care executive and physician champion for VIONE – a national medication deprescribing project that was launched at 59 VA programs across the nation will be presented in a thought provoking, objective, evidence based and legacy oriented format. Highlights include a)success factors b)networking c) effective presentations d) lessons learned e)implementing elements of High Performance Delivery Model (HPDM), f)transforming ideas into actions that help rise and shine g) creating a legacy h)survey readiness h) keeping the main thing the main thing i) understanding others and helping others understand you. Making significant contributions in career is important. How the work is captured, projected, made visible as a viable, actionable solution to a critical and significant mission critical problem, strategic engagement with stakeholders, implementation with measurable outcomes, passionate engagement while being an affirmative, non-controversial leader who is eager, willing, available, capable and knowledgeable etc., are some of the other non-negotiable aspects of getting work recognized. Experiences shared include creating VIONE – a medication deprescribing project that won VA USH Shark Tank competition as a Gold Status Fellow with outcomes such as over 95,000 veterans impacted, over 194,000 non-essential medications deprescribed with accomplished cost savings of over 9.6 million US dollars in 4 years. This profound journey taught me the importance of effectively displaying worthy work with global relevance with dignity, credibility, pride, persuasion, leadership, efficiency, teamwork, art of persuasion, ponder, recharge, rejuvenate and start fresh – time and again.

